# Do or Die: HPV E5, E6 and E7 in Cell Death Evasion

**DOI:** 10.3390/pathogens11091027

**Published:** 2022-09-09

**Authors:** Josipa Skelin, Ivan Sabol, Vjekoslav Tomaić

**Affiliations:** Division of Molecular Medicine, Ruđer Bošković Institute, Bijenička cesta 54, 10000 Zagreb, Croatia

**Keywords:** E5, E6, E7, HPV, cervical cancer, apoptosis, autophagy, anoikis, pyroptosis

## Abstract

Human papillomaviruses (HPVs) infect the dividing cells of human epithelia and hijack the cellular replication machinery to ensure their own propagation. In the effort to adapt the cell to suit their own reproductive needs, the virus changes a number of processes, amongst which is the ability of the cell to undergo programmed cell death. Viral infections, forced cell divisions and mutations, which accumulate as a result of uncontrolled proliferation, all trigger one of several cell death pathways. Here, we examine the mechanisms employed by HPVs to ensure the survival of infected cells manipulated into cell cycle progression and proliferation.

## 1. Introduction

Papillomaviruses (*Papillomaviridae*) are a large group of double-stranded small DNA viruses composed of over 400 different species which infect the epithelia of a wide range of hosts, including mammals, birds, reptiles and fish. Of those 400, more than 200 types have been reported to infect humans [1]. Human papillomaviruses (HPVs) are divided into five genera (α, β, γ, µ and ν), based on the open reading frame sequence coding for the L1 capsid protein [2]. The α and β genera are known to cause health problems in humans and, therefore, are the most extensively studied [3,4].

α-HPVs infect the basal layer of actively dividing mucosal epithelia, which can be found surrounding various cavities of the human body. Based on their ability to cause cancer, α-HPVs are classified either as high-risk (HR), which have oncogenic potential, or as low-risk (LR), which mostly cause self-limiting benign warts [5]. Infection by HR HPVs can lead to the malignant transformation of infected cells during prolonged infection, while LR HPVs, such as HPV-6 and -11, cannot lead to this [6]. The International Agency for Research on Cancer (IARC) classified 14 HPV types as class 1 carcinogens, the most prevalent being HPV-16 and HPV-18, with six other types (HPV-31, -33, -35, -45, -52 and -58) occurring at significant frequencies, depending on the geographical regions [5]. HR HPVs are responsible for 99% of all cervical cancer (CC) cases, as well as 30–90% of other anogenital cancers and 40–60% head-and-neck squamous cell carcinomas (HNSCC) [7,8,9].

HPV replication and virion production depend exclusively on the differentiation processes of keratinocytes, which are the primary target cells of HPVs. The virus intervenes in this process in order to prolong the proliferation of infected cells, and this inevitably triggers the signaling cascade that leads to apoptosis. Likewise, uncontrolled cell division leads to contact inhibition and senescence, i.e., anoikis, while the viral infection itself triggers autophagy. For these reasons, HPVs have evolved various strategies to evade programmed cell death and ensure the continued progression of HPV infection, resulting in the propagation of new virions. In this review, we will explore the intricacies of these strategies and all the ways that HPVs use their limited number of proteins to modify cell death signaling pathways to their benefit.

## 2. HPV Genome Organization and Life Cycle

HPV genome is organized into the early (E) region, the late (L) region and the long control region (LCR), where the origin of DNA replication is located. The proteins encoded by the E genes are expressed from the early promoter and they include E1, E2, E4, E5, E6 and E7. E1 and E2 serve to initiate and regulate viral DNA replication and transcription; E4 facilitates viral gene amplification, viral particle assembly, and release; while E5, E6 and E7 are oncogenes. E5 is considered to be the minor oncoprotein in humans as it is unable to cause cellular transformation; it usually serves non-essential, but pro-carcinogenic roles in HR HPVs. E6 and E7 are the two major oncoproteins, whose joint action promotes the uncontrolled cell cycle progression and proliferation of infected cells. The precise roles and functions of the three aforementioned viral oncoproteins will be discussed later in this review. L1 and L2, which are expressed under the control of the late promoter, are the major and minor capsid proteins, respectively, which form the icosahedral capsid of the virus, ensuring proper viral packaging and assembly [10,11].

In the cervix, the principal and most thoroughly studied anatomical site of infection, HPV seems to invade the epithelium through micro traumas. After entering, it infects undifferentiated and actively dividing cells in the basal layer at the squamo-columnar junction, as these are the only replicatively active cells [12]. HPV does not encode the genes required for DNA replication, so E1 and E2 hijack the host DNA replication machinery and quickly amplify the viral genome to about 50–100 copies per cell [13]. As the basal cells divide under the control of E6 and E7, viral genomes are equally distributed between daughter cells. E2 keeps the expression of E6 and E7 low by binding to their promoter and thus blocking the access to transcription factors until the cells begin the process of differentiation and migration up through the suprabasal layers of the epithelium [14]. As this occurs, E6 and E7 expression is upregulated in order to keep the infected cells in a proliferative state, to expand the replicative compartment and to delay the terminal differentiation of the infected keratinocytes. All this ensures a suitable cellular milieu for viral genome amplification. E5 supports this process, particularly in DNA synthesis and viral genome amplification [15]. In the late phase of the viral life cycle, viral gene expression is amplified and many thousands of viral genomes are produced, supported by the actions of E4 [16]. The minor coat protein (L2) is then produced in the topmost layers of the epithelium, just before the keratinocytes exit the cell cycle. E2 recruits L2 proteins to the regions of replication, after which the major coat protein (L1) is synthesized. Because keratinocytes in the topmost layer are now allowed to enter terminal differentiation, they lose mitochondrial oxidative phosphorylation and convert from a reducing to an oxidizing environment, which allows the formation of disulfide bonds between L1 proteins. Viral DNA is packaged into virions, which are then released without cell lysis, as the cells are shed from the cornified epithelium [17].

HPV infection usually resolves within two years, but around 10% of infected women develop a persistent infection [18] and, of these, a portion will develop cancer. Cellular transformation is caused by increased E6 and E7 activities, which are, in turn, mostly caused by HPV genome integration into the host genome. HPV integration was found in 83% of cervical cancers, 69% of HPV-positive head-and-neck cancers, and 45% of HPV-positive anal cancers, with integration correlating with increased cancer invasiveness [19]. The viral genome can integrate as a single genome into cellular DNA or as multiple head-to-tail (concatemeric) repeats [18]. Although, the majority of HPV-induced cancers contain integrated viral DNA, a minority carry only episomal viral DNA, or a mix of both. Interestingly, even in the cases where the genome is not integrated, HPV DNA was found to have acquired genetic or epigenetic changes that result in dysregulated E6/E7 expression [18]. The circumstances that lead to viral genome integration are still mostly unknown. It occurs at random places in the host genome and leads to the loss of all viral genes, except E6 and E7. With integration, the expression of E6 and E7 falls under the control of cellular promoters and it becomes dependent on the cell cycle as the E2 gene is lost. The E5 gene is, accordingly, mostly lost in this process, but in some cases E5 was found to be expressed in high grade cervical lesions and HPV-driven cancers, most likely due to the presence of an episomal form of the virus or concatameric viral genome integration [20,21,22]. Viral genome integration is not a part of the normal viral life cycle, as it causes the loss of genes needed for completion of the synthesis of viral particles. Once the HPV genome is integrated, the infection becomes abortive and no more virions are created or released [11].

## 3. Apoptosis

Apoptosis is a distinct form of programmed cell death and a highly controlled process involved in maintaining homeostasis during a variety of events, such as differentiation, tissue development, infection and injury [23]. Cells that undergo apoptosis cease to grow and divide. Instead of normal functions they start a series of molecular events that lead to their deaths without the spillage of cellular contents into the surrounding area. We can differentiate between two different apoptosis pathways–the intrinsic, dependent on the release of cytochrome C from the mitochondria, and the extrinsic, which is initiated by the binding of the appropriate ligands to the receptors on the cell surface [23].

The intrinsic or mitochondrial pathway of apoptosis is usually triggered when the cell has suffered damage or when the pro-survival signals from its environment are ablated. In these cases, the mitochondrial permeability transition pore opens and pro-apoptotic proteins leak into the cytoplasm [24]. Cytochrome C then induces conformational changes in the APAF1 protein, which exposes its caspase recruitment domain (CARD domain) and oligomerization domains. This allows APAF1 to oligomerize, forming an apoptosome, which has multiple caspase 9-binding sites (CARD domains) [25]. Binding to procaspase 9 leads to its activation, and activated caspase 9 can then activate procaspase 3 and, with it, the caspase signaling cascade [23].

**Figure 1:**
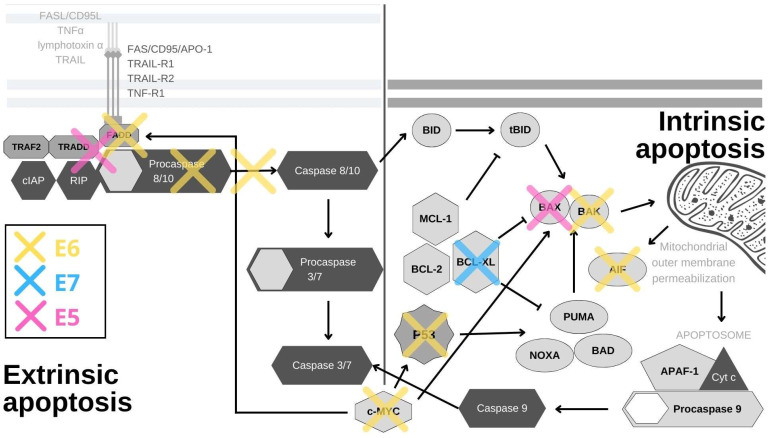
HPV oncoproteins interfere with apoptosis through a variety of mechanisms. E6 (yellow) induces the proteasomal inactivation of p53, the internal detector of cellular stress, and by this activity, it impacts the expression of survivin, YY1 and Cdc2. E6 also degrades the pro-apoptotic proteins BAK, AIF and c-MYC, and cooperates with E5 in inhibiting FAS-mediated apoptosis. By inhibiting TNF-triggered apoptosis, E6 effectively abrogates the external apoptotic pathway and by blocking procaspase activation, it stops the downstream signaling. E6 also utilizes its PDZ-binding motif (PBM) to degrade PDZ domain-containing proteins, leading to the disruption of the apoptotic cascade. E7 (blue) abrogates the binding of SIVA-1 to BCL-XL and thereby prevents the neutralization of anti-apoptotic effects of BCL-XL. E5 (pink) impacts the internal and external apoptotic pathways by inducing the degradation of the pro-apoptotic protein BAK, and impairing the signaling from FAS- and TRAIL-ligands.

The external or death receptor pathway of apoptosis is activated when death ligands bind to their appropriate receptors on the cell surface. The most comprehensively characterized death receptors are APO-1/Fas (CD95), TRAIL-R1 and TRAIL-R2, as well as TNFR1, all of which are members of the tumor necrosis factor (TNF) receptor gene superfamily [26]. When their corresponding ligands CD95L, TNFα, lymphotoxin-α and TRAIL bind to the receptors, they initiate receptor trimerization, which brings their death domains into close proximity [27]. This allows the recruitment of adaptor molecules, such as FAS-associated death domain (FADD) or TNF receptor (TNFR)-associated death domain (TRADD) and procaspase-8, resulting in the formation of the death-inducing signaling complex (DISC) [28]. Caspase-8 is activated within the DISC and it is able to activate downstream caspases and initiate the caspase cascade [29]. The internal and external pathways of apoptosis are depicted in Figure 1.

A viral infection can also trigger apoptotic signaling within the cell, which is why various viruses have developed different mechanisms to prevent this, HPV being one of them. The inhibition of apoptosis has some particularly long-term consequences in the case of oncogenic viruses, whose transforming properties are amplified by their modulation of programmed cell death pathways. Interestingly, cancers that arise from these viral actions are more resistant to therapies, and therefore uncovering the precise mechanisms of resistance could open potentially very important avenues for therapeutic actions.

p53, the guardian of the genome, is the principal cellular target of HR HPV E6 oncoproteins (Figure 1). Its role is to respond to cellular stress or DNA damage and trigger cell cycle arrest or apoptosis, which it induces mostly by the transcriptional activation of the pro-apoptotic proteins PUMA and NOXA. These proteins then activate other pro-apoptotic proteins in the Bcl2 family, such as Bax and Bak, to induce mitochondrial instability and caspase activation [30]. In normal cells, p53 expression is kept at low levels by the actions of the E3 ubiquitin ligase MDM2. Upon the activation of the DNA damage response, p53 is phosphorylated, which inhibits its binding to MDM2. This leads to p53 accumulation in cells where it acts as a transcription factor for genes involved in cell cycle arrest or cell death [31]. In HPV-infected cells, E6 forms a ternary complex with p53 and the ubiquitin-ligase E6AP (E6-associated protein), which results in the ubiquitination and subsequent degradation of p53 at the proteasome [32,33]. Interestingly, E6/E6AP complex formation is also crucial for E6 protein stability, as E6 by itself quickly undergoes proteasomal degradation [34,35]. Even though p53 is principally inactivated and degraded by the E6/E6AP complex, its function can also be subdued independently of E6AP in a number of ways [36]. The interaction of E6 and p53 inhibits its binding to DNA, possibly due to the changes in protein conformation [37]. By interacting with p53 and its binding partners BCP/p300, hADA3 and TIP60, E6 abrogates the transactivation of p53-responsive genes [38,39,40]. Furthermore, E6 sequesters p53 in the cytoplasm, where p53 cannot exert its influence [37]. Other, often overlooked, tactics for p53 abrogation are post-translational protein and epigenetic modifications. E6 inhibits the p300-mediated acetylation of both p53 and histones, in this way repressing the activation of p53 target genes, among which is p53 itself [37,38,41]. E6 also blocks p53 phosphorylation, which prevents its binding to the p21 promoter, further restraining its function [42]. Conversely, overexpression of p53 was found to be a predictive marker for cisplatin therapy resistance in cases of cervical cancer and mutations in the p53 gene were shown to be connected to radiotherapy resistance in HNSCC [43,44]. In addition, the presence of p53 in the cell is impacted by the upregulation of the YY1 transcription factor [45]. The overexpression of YY1 increases p53 ubiquitination and degradation, and YY1 is upregulated in cervical cancer tissues, which makes it a possible biomarker and a new drug target [45,46]. While the precise mechanism behind this is unknown, YY1 can protect cells from apoptosis, as its inhibition induces p53 activation and cell death in HPV-18 expressing HeLa cells [45]. Mediated by p53 degradation, E6 affects the expression of survivin—a member of the inhibitors of apoptosis (IAP) gene family. E6 was shown to transactivate the survivin promoter, thereby increasing the cell’s resistance to apoptosis [47]. Accordingly, survivin was found to be upregulated in in cervical lesions and could potentially be used as an early marker of cervical carcinogenesis [48]. Surprisingly, in a rare example of an anti-apoptotic effect, E6-mediated p53 degradation leads to the upregulation of Cdc2 and the sensitization of HPV E6-expressing keratinocytes to apoptosis in response to therapeutic agents [49].

In a manner similar to its effect on p53, E6 stimulates E6AP-mediated degradation of BAK and c-MYC proteins (Figure 1). BAK is a pro-apoptotic protein which activates the intrinsic apoptotic program, while c-MYC can trigger the extrinsic and amplify the intrinsic apoptotic pathways [4,50,51]. Interestingly, the *c-MYC* gene is found in the chromosomal region most commonly impacted by HPV genome integration (8q24), and c-myc overexpression was found to correlate with HPV amplification, making it a potential biomarker for cervical cancer [52,53] Another E6 substrate is the apoptosis-inducing factor (AIF), a pro-apoptotic flavoprotein involved in the mitochondrial apoptotic pathway, which E6 also binds and targets for degradation [54]. E6 also abrogates the extrinsic apoptotic pathway by subverting FADD signaling. By binding to the death effector domains of the FADD receptor, E6 accelerates its degradation and inhibits FAS- and TRAIL-mediated apoptosis (Figure 1). There is also evidence that this pathway is additionally inhibited in keratinocytes expressing HPV-16 oncoproteins, as a result of the downregulation and cytoplasmic sequestration of the TNF receptor 1, combined with a shift towards the expression of a type 2 TNF receptor, which has a weaker response to TNF-α stimuli [55]. Additionally, E6 was recently found to interact with DAXX, another protein involved in FAS-mediated apoptosis, and, in this manner, to decrease the rate of apoptosis. E6 also inhibits TNF-triggered extrinsic apoptosis by binding TNF R1 [56,57,58,59]. In addition to directly inhibiting cell death, E6 can also activate pro-survival pathways. External stimuli, such as pro-inflammatory cytokines, can activate the signal transducer and activator of transcription 3 (STAT3) transcription factor, which has been found to be essential for the survival of cervical cancer cells. E6 activates STAT3 by inducing the expression of the pro-inflammatory cytokine interleukin-6 via the Rac1-NF-kappaB pathway. STAT3 and NF-kappaB co-activation is a specific marker of HPV-positive HNSCC, while STAT3 mRNA detection can be used for cervical lesion screening with great specificity [60,61]. The activation of STAT3 determines the sensitivity of cervical cancer cells to TRAIL-induced apoptosis and can be abrogated by inhibiting the Janus kinase 2 (JAK2), which phosphorylates STAT3 [62,63].

PDZ (PSD95/Dlg/ZO-1) domains are protein–protein interaction modules, 80–90 amino acids in length, that can be found on a large number of proteins. Some of these PDZ domain-containing proteins are well-described targets of the E6/E6AP complex, while some are degraded by E6 independently of E6AP [64]. At its extreme C-terminus, HR HPV E6 contains a Class I PDZ-binding motif (x-T/S-x-L/V) (PBM) through which it interacts with PDZ domain-containing proteins and modulates their functions [65,66]. One of these proteins is MAGI-1, whose degradation facilitates the disruption of tight junctions. Interestingly, restoring MAGI-1 expression in E6-containing cells results in the induction of apoptosis and repression of cell proliferation [66,67]. E6 also utilizes its PBM in NF-kappaB activation, which leads to the accumulation of cellular inhibitor of apoptosis protein 2 (cIAP-2) and subsequently to cellular resistance to TNF-induced apoptosis [68].

The impact of E6 on cell death can also be exerted through DNA methylation. Death-associated protein kinase 1 (DAPK1) is a component of the endoplasmic reticulum (ER) stress-response pathway and a regulator of apoptosis and autophagy [69]. E6 downregulates its expression by inducing the methylation of its promoter, which could be the possible reason behind its inactivation in cervical cancer cells [70,71]. The precise mechanism for this is unknown, but one possible way could be through the upregulation of DNA methyltransferases (DNMTs) [72]. p53 and SP1 bind the DNMT1 promoter and suppress the transcription of the gene. Thus, when E6 degrades and inactivates p53, it effectively increases the expression of DNMT1 [72]. Nevertheless, DNMT1 was found not to have an impact on DAPK1 promoter methylation in lymphoma cells, but a similar mechanism involving other DNMTs is likely to be involved [73]. DAPK1 promoter hypermethylation was found to be correlated with the increasing severity of neoplasia in cervical biopsies and it could potentially be used as a biomarker [74,75]. Furthermore, E6 can bind to the death effector domain of procaspase-8, inhibit its activation and also induce its degradation (Figure 1). Through acting on both receptors and effectors, E6 decreases the activation of both caspase 3 and 8 [56,58]. The two major HPV oncoproteins, E6 and E7, have also been found to upregulate the UHRF1 protein (ubiquitin-like containing PHD and RING finger domain 1) in cells isolated from the early stages of cervical cancer [76]. This protein has been found to bind and regulate DNA methylation in order to regulate gene expression. UHRF1 upregulation by E6/E7 leads to downregulation of gelsolin and UbcH8, which results in inhibition of cell death [76,77].

The role of E7 in apoptosis resistance is much less clearly defined than that of E6. The main cellular target of E7 is the tumor suppressor retinoblastoma protein (pRb). It is normally bound to E2F and this repression complex blocks G1/S cell cycle progression. E7 hijacks the cullin-2 ubiquitin ligase complex and uses it to induce the proteasomal degradation of pRb. The E2F transcription factor is then released and cell cycle progression is ensured [78]. In a similar manner, E7 also degrades the other two pocket proteins, p107 and p130, leading to E2F4 and E2F5 release, respectively, and in this way also further promotes cell cycle progression [3]. Unregulated cell cycle progression would normally trigger apoptosis through a p53-dependent pathway, but, considering that p53 is degraded or inactivated by E6, apoptosis is prevented in cells expressing both oncogenes [79].

Interestingly, E7 was found to sensitize cells to apoptosis, when the Fas receptor was stimulated [80]. Additionally, keratinocytes expressing E7 were found to be more prone to TNF-α and TRAIL-mediated apoptosis, possibly due to the lack of E6 expression, although this is contested by research showing that E7 expression in normal fibroblasts protects the cells from TNF-α and FAS-induced cell death [81,82]. E7 was also found to inhibit apoptosis by enhancing the degradation of a pro-apoptotic protein, insulin-like growth factor-binding protein-3 (IGFBP-3) [83]. In addition, E7 upregulates the expression of catalase and NF-kappaB, through which it conveys the resistance to H2O2-induced cell death [84]. The expression of the cancerous inhibitor of protein phosphatase 2A (CIP2A), an oncoprotein previously implicated in cell proliferation, senescence and apoptosis resistance, was also found to be upregulated by E7 and this upregulation can be used for screening and diagnosis [85]. Furthermore, E7 associates with the pro-apoptotic protein SIVA-1 and disrupts its binding to BCL-XL. The interaction between SIVA-1 and BCL-XL is important for neutralizing the anti-apoptotic effects of BCL-XL, so E7 effectively decreases the rate of apoptosis by abrogating this interaction (Figure 1) [86]. Among other binding partners, E7 also binds histone deacetylases (HDACs) and this interaction was found to contribute to apoptosis resistance, as inhibiting HDACs in E7-expressing cells sensitizes cells to apoptosis and even causes apoptosis by disrupting the mitochondrial transmembrane potential [87,88,89].

E5 has also been implicated in apoptosis modulation and, similarly to E6, it has been found to induce degradation of a pro-apoptotic Bcl-2 family member, in this case Bax, through increased ubiquitination (Figure 1) [90]. During HPV infection, E5 enhances the activation of EGFR, and the PI3K-AKT and ERK1/2 MAPK signaling pathways, all of which have been found to be major survival components [91]. E5 also prevents the external pathway of apoptosis by impairing the formation of DISC via the FAS or TRAIL ligands (Figure 1) [92]. E5 also represses the expression of 179 components of the endoplasmic reticulum that are involved in stress-response pathways [93]. Taking all this together, E5 does not seem to have a decisive role in the evasion of apoptosis, but certainly has an impact on it through several different mechanisms. As E5 expression is found only in a small portion of HPV-induced cancers, its significance for apoptosis evasion in malignancies is not yet clear [94,95].

Since apoptosis is the principal method of programed cell death, HPVs have developed a number of different ways to evade it and ensure the survival of infected cells, the most important of which are summarized in Figure 1. The three viral oncoproteins each control different aspects of this process in order to secure the completion of the viral life cycle and the release of viral progeny. Of the three, and based on the sheer number of interacting partners and signaling pathways it impacts, E6 seems to be the most important for subverting apoptosis. In the general scheme of HPV infections and carcinogenesis, E6 ensures the survival of infected cells, while E7 promotes proliferation and E5 is thought to play a supportive role. Nevertheless, in regards to cell death evasion, HPV oncoproteins sometimes overlap in function. Unfortunately, after the integration of the viral genome into the host cell DNA, which leads to the uncontrolled expression of viral E6 and E7 oncoproteins, these evasion strategies become the major drivers of cancer resistance to therapy.

## 4. Autophagy

**Figure 2:**
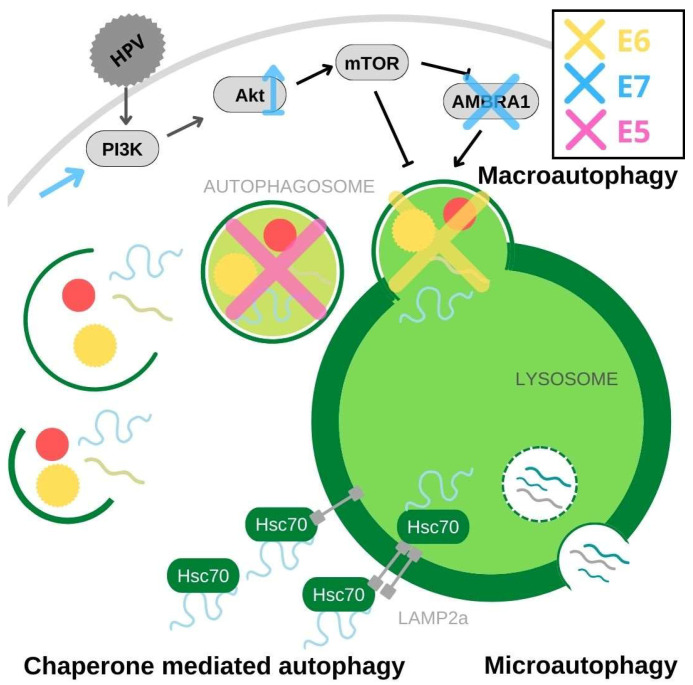
HPV oncoproteins impact different aspects of autophagy. HPV binding to the cell surface induces PI3K activation, which leads to mTOR expression and autophagy inhibition. E7 (blue) also activates PI3K and increases Akt stability. Conversely, E7 also causes degradation of AMBRA1, a negative regulator of autophagy. E6 (yellow) and E5 (pink) inhibit autophagy through p53 degradation. E6 also inhibits the fusion of the autophagosome to the lysosome and E5 downregulates the genes necessary for autophagosome turnover and assembly.

Autophagy is a conserved catabolic process during which a cell degrades its intracellular components as a response to stress, degeneration, infection or carcinogenic changes. During autophagy, materials that are destined for degradation, such as long-lived proteins, organelles or pathogens, are encapsulated in a compartment which is then fused with a lysosome and its contents degraded [96]. Based on the way the materials are encapsulated and delivered for degradation, we recognize chaperone-mediated autophagy, microautophagy, and macroautophagy (Figure 2). Chaperone-mediated autophagy utilizes molecular chaperones which recognize specific sequences in unfolded cytosolic proteins. They then bind to these proteins and shuttle them to the lysosome by interacting with a lysosome-associated membrane protein (LAMP) type 2A [97]. Microautophagy is a process used by cells to digest small volumes of cytosol. During microautophagy, the membrane of the lysosome invaginates and encapsulates the material that needs to be degraded [98]. The most common and well-studied form of autophagy is macroautophagy, during which large portions of cytoplasm and its contents are engulfed in an organelle called the autophagosome. This fuses with the lysosome to form an autolysosome, in which degradation occurs and useful macromolecules are recycled. Overall, autophagy is an incredibly complex mechanism involved in various physiological processes and its detailed mechanisms are comprehensively reviewed in Khandia et al., 2019 [96].

During viral infections, autophagy is crucial for mounting an innate defense against invaders, as the process recognizes and aims to degrade viral particles within the cell, as well as inducing the innate immune response by promoting inflammation, antigen presentation and cytokine response [99]. As a result of the constant host–pathogen co-evolution, some viruses have developed mechanisms to evade autophagy or even to use it to their advantage, with HPV belonging to the former group [100]. The inhibition of autophagy begins immediately after the binding of the viral particle to the heparan sulfate proteoglycans on the keratinocyte surface [101]. This binding activates the PI3K-Akt-mTOR signaling pathway and, with mTOR being a negative regulator of autophagy, it inhibits the cellular process, ensuring the uninterrupted trafficking of viral particles into the cell (Figure 2) [101]. Similarly, small molecule inhibition of autophagy at different steps has been shown to increase the infectivity of HPV-16 [102]. Inhibition of autophagy during early viral infection is dependent on the activity of the L1 and L2 capsid proteins rather than the HPV oncoproteins, but this nevertheless highlights the importance of inhibiting autophagy to allow successful viral infection. However, as HPV oncoproteins are successively expressed, they become involved in autophagy inhibition and sustain this activity throughout the infection. Indeed, a microarray analysis has shown that silencing E6/E7 induces autophagy in keratinocyte cell lines containing either episomal or integrated HPV genomes [103]. HPV 16 oncoproteins expressed in primary human keratinocytes have been found to inhibit autophagy by inhibiting the fusion of autophagosomes and lysosomes, thereby effectively preventing the degradation of encapsulated targets (Figure 2). This effect was found to be mostly due to E6/E6AP complex-dependent degradation of p53 [104]. Considering that E6 stability mostly depends on its interacting partner E6AP [34,105], and that chaperone-mediated autophagy could be the possible mechanism for E6 protein turnover in the absence of E6AP, it would be interesting to further investigate the role of autophagy in E6 oncoprotein stability.

There is contradictory evidence regarding the role of HPV E7 oncoproteins in evasion of autophagy. E7 contributes to autophagy inhibition by mediating the degradation of AMBRA1, a positive regulator of autophagy and a negative regulator of proliferation (Figure 2) [106]. Nevertheless, there is also evidence of E7’s pro-autophagic effects. Expressing E7 alone in normal human keratinocytes leads to the increased formation of autophagosomes and sensitizes cells to cell death in response to growth-factor deprivation [107]. Furthermore, E7 also activates the aforementioned PI3K-Akt-mTOR signaling pathway through pRb degradation and prolongs signaling through the PI3K-Akt axis by binding PP2A, a phosphatase important for Akt dephosphorylation. Inhibiting Akt dephosphorylation prevents the inactivation of the pathway and ensures the expression of the negative regulator of autophagy, mTOR (Figure 2) [108,109]. High levels of phosphorylated m-TOR expression in HPV-related cancers can serve as a prognostic marker for poor chemotherapy response and overall patient survival, and mTOR inhibitors, such as rapamycin, are currently being tested as potential therapeutic targets [110,111,112]. E7-mediated degradation of pRb and p130 also results in a release of E2F5, which leads to the activation of DRP1. The activated DRP1 translocates to the mitochondria, where it causes mitochondrial fission and subsequent lethal mitophagy, a form of macroautophagy caused by mitochondrial degradation [113].

E5 attenuates autophagy by down-regulation of the genes important for the assembly and turn-over of autophagosomes in keratinocyte growth factor- and serum-deprived cells (Figure 2) [114]. This process is p53-dependent, as it is essential for the transcription of the genes in question (*BECLIN 1, ATG5, LC3, ULK2, ATG4a, and ATG7*) [114]. E5 seems to use independent mechanisms to abrogate p53 function, but the details of this mechanism are currently unknown [114]. As E5 is mostly expressed in the basal and suprabasal layers of infected epithelia, where cells start their differentiation process, this indicates the importance of auxiliary mechanisms of autophagy resistance in this crucial period of infection.

Little is still known about the influence of HPV oncoproteins on autophagy and about the mechanistic details of this process (Figure 2), but more is continuously being discovered. HPVs terminate different modes of autophagy within cells in order to ensure their own survival, since autophagy could be detrimental during early infection. However, during persistent infection this inhibition inadvertently has consequences that promote cell transformation and tumorigenesis. Autophagy contributes to the ability of the cell and the organism to limit viral replication, enhance the immune response and eliminate transformed cells; thereby protecting the organism from infection. Unfortunately, the same processes can also help cancer cells recover from metabolic stresses caused by chemotherapy and, as a consequence, can contribute to chemotherapy resistance and cancer recurrence. In the case of cervical cancer, autophagy seems to have an anticarcinogenic role. The expression of autophagy-related markers was found to be reduced in cervical and anal cancers, but the precise significance and prognostic value of this is not yet clear [115,116,117].

## 5. Anoikis and Pyroptosis

Anoikis is a form of orderly cell death induced by detachment of cells from the extracellular matrix. It is a crucial mechanism that serves to prevent adherent-independent cell growth and is, therefore, critical for preventing metastatic colonization [118]. Notch1 activation in HPV E6/E7-expressing immortalized epithelial cells was found to convey resistance to cell death in response to matrix withdrawal, and this resistance is mediated through PKB/Akt signaling [119]. HPV E6/E7 was also shown to induce Nurr-1 expression, which promotes anchorage-independent growth, cell proliferation and migration of HPV-positive cervical cancer cells and cell lines [120]. Furthermore, PIK3CA was also demonstrated to be upregulated in cervical carcinomas and frequently mutated in HPV-associated oropharyngeal squamous cell carcinoma [121,122]. This protein is a positive regulator of the PI3K/Akt pathway, and blocking its activity reduced cell viability and anchorage-independent growth in HPV immortalized cells [121]. HPV oncoproteins do not interact directly with PIK3CA: neither with its promoters nor with its interacting partners; but are thought to promote activating mutagenesis of the PIK3CA gene. Due to their mechanisms of action, HPV oncoproteins are thought to drive cell mutagenesis and a number of genes, such as PIK3CA, PTEN, HLA-A/B and TGF-β, are commonly found to be somatically mutated in HPV-positive cancers [123]. Another potentially important gene dysregulated in HPV-associated cancers is SORBS2, which was revealed by microarray analysis to be downregulated in cervical premalignant lesions and HNSCCs [124,125]. The expression of SORBS2 suppresses the metastasis of a number of cancers, and its ectopic expression in cells in vitro results in a significant reduction in anchorage-independent growth and colony formation [124,126,127]. Similarly, to PIK3CA, no direct interaction between HPV oncoproteins and SORBS2 has been found, so the mechanism by which HPV impacts SORBS2 expression still remains to be elucidated. Caveolin-1, a component of caveolae, whose role in tumor progression depends on the cellular context, is consistently found to be downregulated in cervical cancer-derived cell lines, and this reduction is dependent on E6-induced p53 degradation. In HNSCCs, the lack of Calveolin-1 leads to increased epithelial-mesenchymal transition [128], while restoring expression of calveolin-1 abrogates anoikis resistance [129]. Interestingly, E5 was found to upregulate caveolin-1, and this upregulation is considered to be a possible mechanism for immune evasion during productive viral infections [130]. Nevertheless, considering that HPV-induced cancers predominantly express E6/E7 oncoproteins, this upregulation is most probably not significant in the context of carcinogenesis. In addition, HPV E6 PBM-PDZ interactions appear to be also significant for viral regulation of anoikis. E6 utilizes its PBM to interact with and induce the degradation of a PDZ-domain containing protein PTPN13, which is implicated in several cell survival pathways. This ablation results anchorage-independent growth and in resistance to anoikis, mediated through Ras signaling [131]. In addition, E7-mediated degradation of tyrosine phosphatase PTPN14, a potential tumor suppressor, was also shown to influence anoikis resistance. The ubiquitin ligase UBR4/p600 was found to be required for this process, although the precise mechanism is still unknown [78,132,133]. The interaction between E7 and UBR4/p600 has been shown to cause anchorage-independent growth, but whether this is due solely to PTPN14, or if there are other proteins involved in the process, remains to be elucidated. Anchorage-independent growth is a prerequisite for metastatic progression of HPV-related cancers and uncovering details about this process is, therefore, the key to stopping it.

Pyroptosis or caspase-1-dependent cell death, is an inflammatory cell death process usually triggered by microbial infections or pathological stimuli. Pyroptotic cell death results in the controlled rupture of the plasma-membrane and the release of cellular contents, which then triggers a rapid immune response [134]. HPV-infected cervical cancer cells were shown to evade pyroptosis by upregulating SIRT1, which represses the transcription of the AIM2 gene, important for the recruitment of procaspase-1 to the inflammasome [135]. Decreased procaspase recruitment to the inflammasome means that less procaspase is activated to caspase and that, consequently, the pyroptotic response is reduced. This SIRT1 overexpression was found to be correlated with the progression of cervical lesions towards development of SCC, which makes it a potential biomarker [136]. Interestingly, inhibiting SIRT1 expression restores pyroptosis, not only in SIRT1 silenced cells, but also in their neighboring cells, and this process is mediated by extracellular vesicles carrying AIM2 proteins [135]. HPV E7 can also inhibit pyroptosis caused by the presence of dsDNA, by utilizing the E3 ubiquitin ligase TRIM21 to degrade the IFI16 inflammasome [137]. Pyroptotic cell death can trigger auxiliary inflammatory processes and immune responses, particularly in response to intracellular pathogens such as HPV, which is why HPVs have developed mechanisms for pyroptosis evasion. Likewise, finding novel ways to restart the process could circumvent the HPV-induced blockade of the immune response and thereby facilitate viral clearance.

## 6. Conclusions

Programmed cell death serves to maintain cellular and tissue homeostasis, and to protect the organism from transformed or infected cells. A number of pathogens, including HPVs, have therefore developed ways to subvert these mechanisms to ensure their own survival. Unfortunately for the infected individual, cell death evasion also allows the continual propagation of damaged or transformed cells. As described here, the primary mediators of HPV-induced cell death resistance are its oncogenes E5, E6 and E7, whose different mechanisms complement and support each other in this process. These oncoproteins impact several signaling pathways indispensable for programmed cell death. They degrade members of the Bcl2 pro-apoptotic family and PDZ-domain containing proteins, while they upregulate anti-apoptotic proteins, such as BAK, c-MYC and SIRT1. Importantly, E6 degrades the principal tumor suppressor p53 and, through this process, it impacts a number of targets crucial for cell death and survival. Additionally, HPV oncoproteins enhance the activity of the survival pathways such as PI3K/PKB/AKT and ERK1/2 MAPK, while they inhibit the external pathways of apoptosis mediated by FAS- and TRAIL-ligands. It is clear that there are still many unknown aspects and contrasting findings in the field of HPV cell death inhibition, but analyzing and elucidating these mechanisms could potentially uncover new protein targets, or avenues that could create a basis for therapeutic action against HPV-driven malignancies.
